# 

**Published:** 1902-03

**Authors:** 


### CONTENTS.

ORIGINAL ARTICLES:
- Anatomo-Pathological Study of Ringbone and Spavin. By S. J. J. HARGER, D.V.M. 133
- The Comparative Virulence of the Tubercle Bacillus from Human and Bovine Sources. By MAZYCK P. RAVENEL, M.D. . . . . . . . . . . 138
- The Horse's Foot. By JAMES T. McDONOUGH, V.S. . . . . . . . . . 156

DEPARTMENT OF CANINE, FELINE AND AVIAN MEDICINE AND SURGERY . 160

ABSTRACTS FROM FOREIGN JOURNALS . . . . . . . . . . . . . 164

REPORTS OF CASES:

- Rabies. By W. L. BELL, D.V.S. . . . . . . . 170

- Acute Œdema of the Tongue. By ORR GRAHAM, V.S. . . . [illegible]

EDITORIALS:

- Some Association Work . . . . . . . . . . . . . 175

- Columella's Proposition . . . . . . . . . . . . . 175

- Massachusetts' New Bureau . . . . . . . . . . . . 176

- Secretaryships . . . . . . . . . . . . . . 176

NECROLOGY . . . . . . . . . . . . . . . . . 177

COMMUNICATIONS—CORRESPONDENCE—SELECTION—AMONG THE COLLEGES—MATTERS OF INTEREST TO YOU—SOCIETY PROCEEDINGS—PERSONALS 179-197

VETERINARY REGISTER—PENNSYLVANIA (*continued*) . . . . . . . . 199

